# Indole-3-carbinol attenuates DNCB-induced atopic dermatitis in mice by reducing inflammation and IL-4/IL-13 expression

**DOI:** 10.17305/bb.2026.14424

**Published:** 2026-07-02

**Authors:** Rabbia Kalim, Arham Shabbir, Hafsa Nasr, Aisha Mobashar, Abdulelah Alhazza, Hamoud Alotaibi, Mona Al Hamod, Saleh Alfuraih, Ali F Almutairy, Sulaiman M Alnasser, Waad Alrohily, Ashfaq Ahmad

**Affiliations:** 1 Department of Pharmacology, Institute of Pharmacy, Faculty of Pharmaceutical and Allied Health Sciences, Lahore College for Women University, Lahore, Pakistan; 2 Faculty of Pharmacy, The University of Lahore, Lahore, Pakistan; 3 Department of Pharmaceutics, Faculty of Pharmacy, Northern Border University, Rafha, Saudi Arabia; 4 Department of Pharmacology and Toxicology, College of Pharmacy, Northern Border University, Rafha, Saudi Arabia; 5 Department of Pharmacology and Toxicology, College of Pharmacy, Qassim University, Buraydah, Saudi Arabia; 6 Department of Pharmacy Practice, College of Pharmacy, Taibah University, Medina, Saudi Arabia; 7 Department of Pharmacy Practice, College of Pharmacy, University of Hafr Al Batin, Hafr Al Batin, Saudi Arabia

**Keywords:** Indole-3-carbinol, atopic dermatitis, interleukin-4, interleukin-13, inflammation, immunomodulatory

## Abstract

Atopic dermatitis (AD), commonly known as atopic eczema, is a chronic inflammatory skin disease. Indole-3-carbinol (I3C), a phytochemical naturally occurring in *Brassica* vegetables, has been traditionally used in folk medicine for inflammatory and metabolic disorders. This study investigated the immunomodulatory and anti-inflammatory effects of I3C in a mouse model of 1-chloro-2,4-dinitrobenzene (DNCB)-induced atopic dermatitis. Atopic dermatitis was induced by DNCB sensitization on days 0 and 3. Following disease confirmation on day 7, mice received daily oral or topical administrations of I3C or prednisolone from day 7 to day 19. Macroscopic evaluations included dorsal skin and ear thickness measurements. Histopathological changes were assessed using hematoxylin and eosin staining, and hematological parameters were also analyzed. Gene expression of interleukin (IL)-4 and IL-13 in ear tissue was quantified by real-time polymerase chain reaction (RT-qPCR). Computational molecular docking was performed to determine I3C’s affinity for both cytokines. The data indicated that I3C attenuated DNCB-induced AD-like symptoms following both oral and topical administration. Treatment with I3C also resulted in reduced ear thickness. Hematological analysis revealed significantly decreased total leukocyte counts (TLC) and a general downward numerical trend in differential leukocyte count (DLC) parameters in I3C-treated groups. Histopathological evaluation confirmed that I3C ameliorated skin thickening and reduced inflammatory cell infiltration. Furthermore, IL-4 and IL-13 expression levels were decreased in I3C-treated groups. Molecular docking analysis demonstrated moderate binding affinities of −5.1 kcal/mol and −5.3 kcal/mol with IL-4 and IL-13, respectively. In conclusion, findings from this exploratory study suggest that I3C treatment ameliorated DNCB-induced atopic dermatitis in mice, evidenced by reductions in ear thickness and IL-4/IL-13 mRNA expression. Molecular docking analysis demonstrated moderate binding affinities of I3C toward IL-4 and IL-13, providing supportive evidence of potential ligand-protein interactions.

## Introduction

Atopic dermatitis (AD), also known as atopic eczema, is a prevalent and severe inflammatory skin disorder characterized by recurrent pruritic conditions that can manifest from infancy through adulthood [1–3]. Both genetic predisposition and environmental factors significantly contribute to the pathogenesis of AD [4]. Eczematous lesions in AD are associated with characteristic inflammatory symptoms, including erythema, pruritus, swelling, and edema [5]. Patients with severe AD frequently experience intense scratching in affected areas, which can exacerbate itching, lead to fluid extravasation, and predispose to bacterial infections [2]. The visible nature of skin conditions in AD can result in social stigma, further intensifying stress and causing sleep disturbances [6]. AD is also recognized as an allergic disorder, triggered by allergens such as dust mites, pollen, certain foods (e.g., milk, seafood), and animal dander, which induce eczematous lesions [2].

AD is further characterized by an imbalance in the ratio of T-helper 1 (Th1) and T-helper 2 (Th2) lymphocytes. While Th1 immune responses are typically elicited by infections, allergic reactions are primarily mediated by Th2 cellular immune responses [4]. Overactivity of Th2 cells can lead to excessive secretion of interleukins such as IL-4 and IL-13, consequently increasing IgE production by B cells and promoting eosinophil and mast cell infiltration into the skin. Therefore, attenuating Th2 cellular activity is expected to ameliorate AD symptoms [2, 6].

Despite the availability of various treatment options aimed at stabilizing the imbalanced state of AD, their success rates remain low [2]. Initial management strategies for AD should prioritize restoring, repairing, and hydrating the skin barrier [7]. Immunosuppressive drugs, such as corticosteroids, are employed both topically and orally, while antihistamines are used to mitigate AD symptoms [6]. However, these approaches often provide incomplete relief and are associated with adverse effects [2]. Corticosteroid-induced side effects include skin striations, visible stretch marks, and skin thinning, whereas antihistamines can cause drowsiness and blurred vision [5, 6]. Consequently, researchers continue to explore alternative therapeutic strategies, with a growing focus on natural compounds [5].

Indole-3-carbinol (I3C) is a naturally occurring phytochemical found in cruciferous vegetables of the *Brassica* genus, including cabbage, mustard, and radish. *Brassica* species have a history of use in traditional medicine for managing various inflammatory conditions, and their phytochemicals have garnered significant interest due to their anti-inflammatory and immunomodulatory properties [8, 9]. Numerous studies have identified I3C as a key active component responsible for the biological effects of these vegetables. I3C exhibits both anti-inflammatory and antioxidant properties and has demonstrated beneficial effects in experimental models of arthritis and obesity [9, 10]. Recent research has confirmed its broad therapeutic potential in various cancers, showing inhibition of experimentally induced carcinogenesis in the cervix, colon, lung, liver, mammary gland, and skin in mouse and rat models [10]. It has also exhibited gastroprotective effects in different clinical conditions [11].

I3C possesses diverse potential therapeutic activities, and a recent study demonstrated its protective effects in experimental AD [12]. Nevertheless, further evidence is required regarding the comparative efficacy of oral vs topical administration of I3C and their specific impact on key Th2-associated cytokines implicated in AD pathogenesis. Therefore, the present study aimed to evaluate the therapeutic effects of orally and topically administered I3C in a DNCB-induced mouse model of AD. This evaluation was conducted through the assessment of ear and dorsal skin lesions, hematological parameters, histopathological alterations, IL-4 and IL-13 mRNA expression, and supportive molecular docking analysis.

## Materials and methods

### Animals

The study utilized 36 BALB/c mice, equally divided by sex, aged 6–8 weeks, with body weights ranging from 28 to 35 g. Inclusion criteria specified healthy adult mice within the designated weight range. Animals exhibiting signs of illness, injury, or abnormal behavior prior to study initiation were to be excluded; however, no animals met the exclusion criteria, and all animals completed the study. Animals were monitored daily throughout the study for general health status, mobility, food and water intake, and signs of overt distress. No animals displayed severe clinical signs necessitating early removal from the study, and all animals completed the experimental protocol. A 7-day acclimatization period preceded the experiment. Mice were randomly allocated into six groups using a lottery method, with each group comprising three male and three female animals. Formal allocation concealment was not implemented. Standard environmental conditions were maintained, including controlled room temperature (23 ± 2 ^∘^C), humidity (50 ± 5%), and a 12-hour light/dark cycle. Mice were provided a standard diet and water *ad libitum*.

Qualitative histopathological assessment was performed by a blinded histopathologist, and clinical scoring was conducted by a blinded independent observer. For other outcome measures, assessments were performed by the same investigator using standardized procedures and predefined scoring criteria; however, formal blinding was not implemented. 

### Induction protocol of AD

DNCB was used to induce AD-like skin lesions following a previously published procedure [5], as depicted in Figure 1. One day prior to disease induction, approximately 1.5 cm × 1.5 cm of dorsal skin on all mice was trimmed with an electric shaving machine. For lesion induction in the diseased and treatment groups, 120 µL of 2% DNCB in acetone/olive oil (3:1) was applied to the dorsal skin of mice on days 0 and 3, while 30 µL of the same solution was applied to the pinna of each ear. On days 6, 9, 12, 15, and 18, mice in the disease control and treatment groups were challenged with 120 µL and 30 µL of 0.5% DNCB in acetone/olive oil (3:1) on the dorsal skin and ears, respectively. Mice were gently restrained for 2–3 min to allow partial absorption.

**Figure 1.**
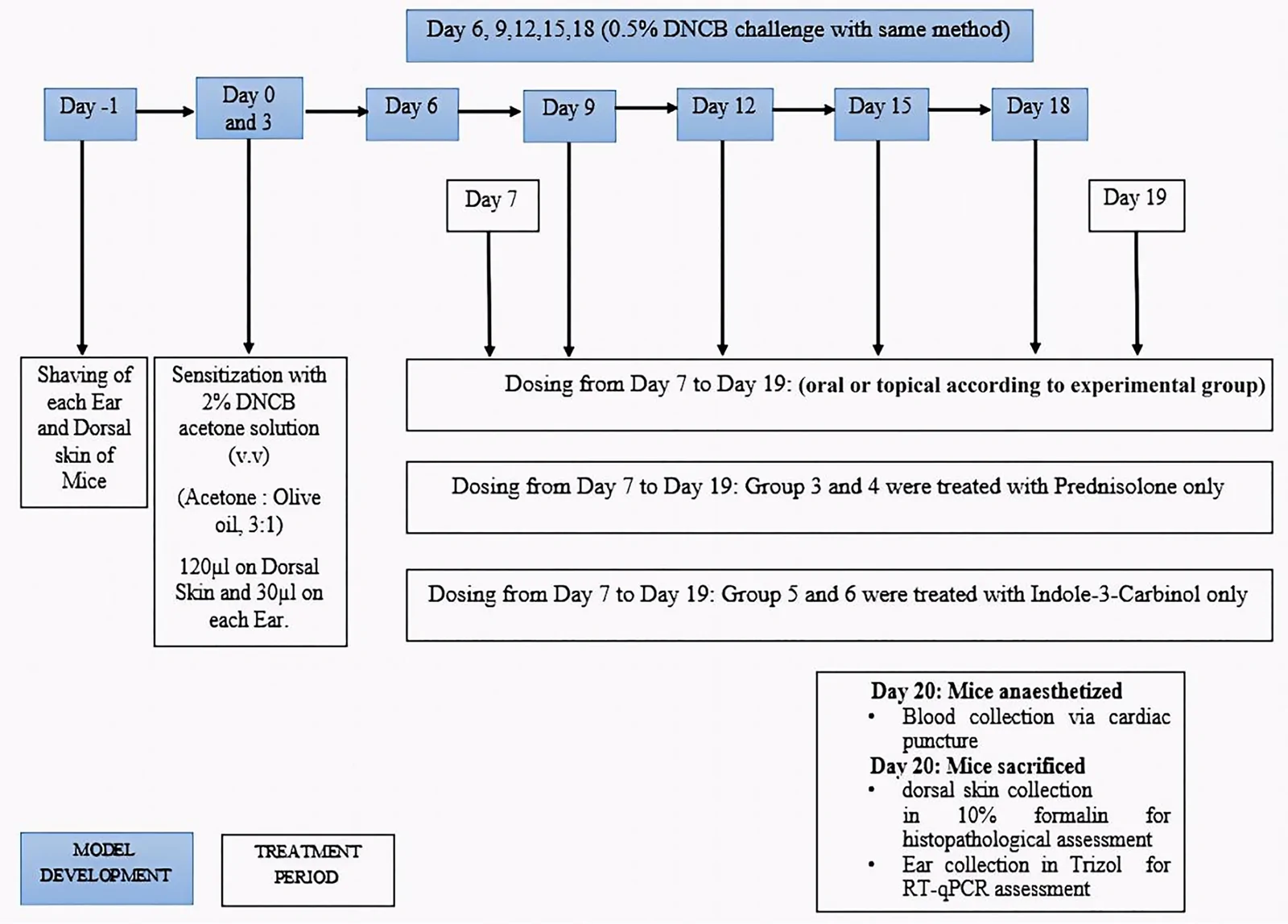
**Experimental timeline for induction and treatment of DNCB-induced atopic dermatitis-like lesions in mice.** Dorsal skin and ears were shaved on day -1. AD-like lesions were induced by sensitization with 2% DNCB in acetone/olive oil on days 0 and 3, followed by repeated challenge with 0.5% DNCB on days 6, 9, 12, 15, and 18. After confirmation of disease induction on day 7, mice received daily treatment from day 7 to day 19 according to the assigned experimental group: vehicle, oral or topical prednisolone, or oral or topical I3C. On day 20, mice were anesthetized, blood was collected by cardiac puncture, dorsal skin was harvested for histopathological assessment, and ear tissue was collected in TRIzol for RT-qPCR analysis. **Abbreviations**: AD, atopic dermatitis; DNCB, 1-chloro-2,4-dinitrobenzene; I3C, indole-3-carbinol; RT-qPCR, quantitative real-time polymerase chain reaction.

### Experimental design

The experimental groups and treatment protocol are summarized in Table 1.

**Table 1:** Experimental groups and treatment protocols

**Experimental group**	**Induction protocol**	**Treatment**	**Route**	**Administered dose**	**Representative amount for a 30 g mouse**	**Treatment regimen**
Control	Animals were sensitized with a vehicle solution composed of acetone and olive oil (3:1) without DNCB application on days 0 and 3. It was followed by repeated challenge on day 6, 9, 12, 15, and 18 with an acetone/olive oil solution (3:1) only.	10% DMSO	Both orally and topically	—		For all groups: Treatment was administered daily for 13 consecutive days (Days 7–19). For topical administration, the stated dose represented a separately calculated body-weight-based dose applied to the dorsal skin and ears (not divided between sites).
Disease Control	Animals were sensitized with 2% DNCB on days 0 and 3, followed by repeated challenge with 0.5% DNCB on days 6, 9, 12, 15, and 18	10% DMSO	Both orally and topically	—		
Standard treatment-I	Same sensitization and challenge protocol as disease Control group	Prednisolone	Oral only	50 mg/kg	1.5 mg	
Standard treatment-II	Same sensitization and challenge protocol as disease Control group	Prednisolone	Topical only	50 mg/kg	1.5 mg	
Test treatment-I	Same sensitization and challenge protocol as disease Control group	I3C	Oral only	100 mg/kg	3.0 mg	
Test treatment-II	Same sensitization and challenge protocol as disease Control group	I3C	Topical only	100 mg/kg	3.0 mg	

**Abbreviations:** DMSO, dimethyl sulfoxide; DNCB, 1-chloro-2,4-dinitrobenzene; I3C, indole-3-carbinol.

Group 1 (Control Group): Mice were induced on days 0 and 3, and subsequently challenged percutaneously on days 6, 9, 12, 15, and 18 on the pinna of each ear and dorsal skin with an acetone/olive oil solution (3:1). During treatment days 7–19, mice received 10% dimethyl sulfoxide (DMSO) orally and topically [4].

Group 2 (Disease Control Group): Mice were induced on days 0 and 3, and subsequently challenged percutaneously on days 6, 9, 12, 15, and 18 with 30 µL of DNCB solution on the pinna of each ear and 120 µL on the dorsal skin. During treatment days 7–19, 10% DMSO was also administered orally and topically [5].

Standard Treatment-I (Oral Prednisolone): Mice were treated orally with prednisolone (50 mg/kg body weight) dissolved in 10% DMSO [13]. Treatment continued for 13 consecutive days, from day 07 to day 19.

Standard Treatment-II (Topical Prednisolone): Mice were treated topically with prednisolone (50 mg/kg body weight) dissolved in 10% DMSO. Treatment continued for 13 consecutive days, from day 07 to day 19. The mixture was spread evenly using a calibrated micropipette on the dorsal skin (120 µL) and ear (30 µL).

Test Treatment-I (Oral I3C): Mice were treated orally with I3C (100 mg/kg body weight), dissolved in 10% DMSO [10]. Treatment continued for 13 consecutive days, from day 07 to day 19.

Test Treatment-II (Topical I3C): Mice were treated topically with I3C (100 mg/kg body weight), dissolved in 10% DMSO. Treatment continued for 13 consecutive days, from day 07 to day 19. The mixture was spread evenly using a calibrated micropipette on the ear (30 µL) and dorsal skin (120 µL).

For oral treatment, I3C and prednisolone were dissolved in 10% DMSO and administered once daily by oral gavage in a fixed volume of 120 µL. For topical treatment, the required dose was calculated according to the body weight of each animal. The calculated amount of drug was dissolved in 120 µL of 10% DMSO for dorsal skin application and in 30 µL of 10% DMSO for ear application. The required amount of I3C or prednisolone was calculated individually according to body weight and administered in fixed application volumes. The calculated daily topical dose was not divided between the dorsal skin and ears. Instead, separate body-weight-based doses were prepared and applied to each site using fixed application volumes of 120 µL for dorsal skin and 30 µL for ears. Consequently, the exact amount of drug applied varied slightly among animals while maintaining the target dose of 100 mg/kg for I3C and 50 mg/kg for prednisolone. The formulations were applied using a calibrated micropipette and evenly spread over the shaved dorsal skin area (approximately 1.5 cm × 1.5 cm) and ears. Following application, animals were gently restrained for a short period to allow absorption of the formulation.

### Sample collection

On day 20, mice were anesthetized by an intraperitoneal injection of a Ketamine/Xylazine cocktail (100/10 mg/kg). Blood samples were collected in EDTA tubes via cardiac puncture. Subsequently, mice were euthanized, and the left ear of each mouse was immersed in TRIzol. Dorsal skin samples were fixed in 10% formalin.

### Macroscopic evaluation of AD scores on the ear and dorsal skin

Clinical severity was assessed using a composite inflammatory score derived from the collective evaluation of erythema, dryness, edema, and inflammation observed on the ears and dorsal skin of mice. Assessments were conducted on days 7 and 19, with scores assigned as follows: 0 = none, 1 = mild, 2 = moderate, and 3 = severe. An independent observer, blinded to the treatment groups, performed clinical scoring using predefined criteria. The assigned scores were subsequently reviewed by the study investigator. Clinical evaluation relied solely on visual inspection; no dermatoscope was employed during assessment.

### Determination of ear thickness

Ear thickness was measured on days 7 and 19 using a digital vernier caliper. Measurements were taken on both ears at a consistent site on the pinna, and the average value for each animal was utilized for statistical analysis. The study investigator performed measurements using standardized procedures. Formal blinding was not implemented for these measurements.

### Histopathological evaluation

Following euthanasia, dorsal skin tissues were collected and preserved in 10% formalin. These tissues were subsequently embedded in paraffin and stained with hematoxylin and eosin (H&E) using previously established methods to observe qualitative histopathological changes [13–15].

### Analysis of hematological parameters

On day 20, blood samples were collected via cardiac puncture into EDTA vacutainers. Total leukocyte count (TLC) and differential leukocyte count (DLC), including neutrophils, lymphocytes, monocytes, and eosinophils, were analyzed in control, diseased, and treatment groups using an automated hematology analyzer [11].

### Analysis of IL-4 and IL-13 expression levels using RT-qPCR

The mRNA expression levels of IL-4 and IL-13 in mouse ear tissues were determined using the following methodology:

### RNA extraction and quantification

Approximately 100 mg of mouse ear tissue was homogenized in 1 mL of TRIzol reagent. Total RNA was then extracted from the homogenized tissue samples following the standard TRIzol method. The total RNA yield was quantified using a NanoDrop spectrophotometer and recorded as the RNA concentration in ng/µL.

### cDNA synthesis via reverse transcription for cDNA

Synthesis, 1000 ng of total RNA per reaction was used with the Reverse Transcription Method. The Thermo Scientific, USA, RevertAid First Strand cDNA Synthesis Kit (#K1622) was employed for cDNA synthesis. The manufacturer’s protocol was followed: 1 µL of Oligo dT18 primer and 6 µL of nuclease-free water were added to the total RNA. The mixture was briefly centrifuged, incubated at 65^∘^C for 5 min, and then immediately placed on ice. Subsequently, 4 µL of 5× reaction buffer, 2 µL of 10 mM deoxynucleotide triphosphate (dNTP) mix, 1 µL of RiboLock RNase Inhibitor (20 U/µL), and 1 µL of RevertAid Moloney murine leukemia virus (M-MuLV) enzyme (200 U/µL) were added to achieve a final volume of 20 µL. The reaction was incubated in a thermocycler at 42^∘^C for 60 min.

### mRNA amplification by qPCR

The cDNA synthesized via reverse transcription served as a template for amplifying IL-4 and IL-13. mRNA expression levels of IL-4 and IL-13 were analyzed using gene-specific primers. The primer sequences utilized were: IL-4 (forward: AGCATCACCCCTGTGTTCCACCC; reverse: TGGGGCAGTCTCCATTGCCA) [4], IL-13 (forward: CCTGGCTCTTGCTTGCCTT; reverse: GGTCTTGTGTGATGTTGCTCA) [5], and glyceraldehyde-3-phosphate dehydrogenase (GAPDH) (forward: ATGTGTCCGTCGTGGATCTGA; reverse: ATGCCTGCTTCACCACCTTCT) [5].

GAPDH was used as an internal control. For each reaction, 2 µL of cDNA was combined with 1 µL of each forward and reverse primer in PCR tubes. 6 µL of Maxima SYBR Green/ROX qPCR Master mix (2×) (#K0221) was added, and the final volume was adjusted to 12 µL with 2 µL of nuclease-free water. The same procedure was followed for the housekeeping gene GAPDH. Tubes were briefly spun for 15 s and placed in a Rotor-Gene Q (Qiagen, USA) thermocycler. qPCR amplification involved an initial denaturation at 95^∘^C, followed by 40 cycles of denaturation at 95^∘^C for 10 s, annealing at 58^∘^C for 20 s, and extension at 72^∘^C for 30 s. All Ct values were recorded. GAPDH served as the reference gene for normalization. No-template controls (NTCs) were included to rule out contamination. Six biological replicates were analyzed, with each sample measured in duplicate as technical replicates, and the mean Ct value was used for analysis. Primer specificity was confirmed by melt-curve analysis. Relative mRNA expression was calculated using the ΔCt method with GAPDH as the reference gene. Values were normalized to the mean of the normal control group (set to 1) to calculate fold change, as previously described [13].

**Figure 2.**
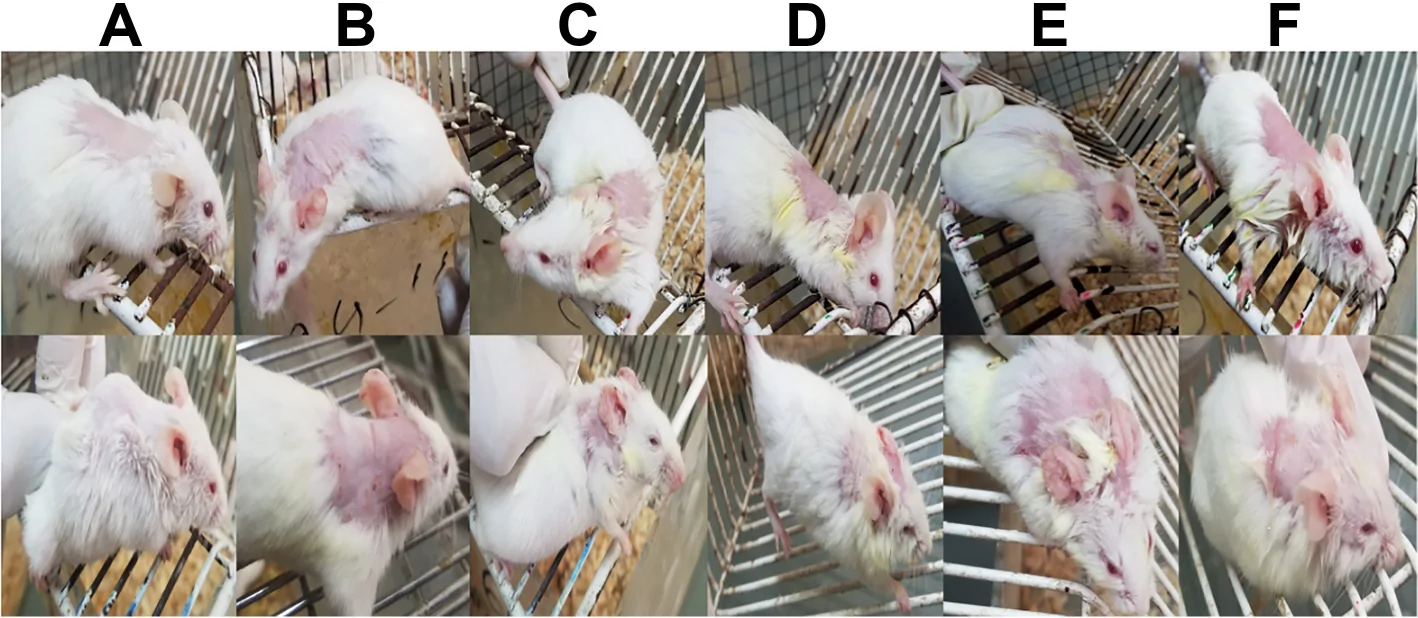
**Representative macroscopic appearance of DNCB-induced atopic dermatitis-like lesions before and after treatment.** Representative photographs of mice on day 7 (upper row; post-induction baseline before treatment initiation) and day 19 (lower row; after daily treatment from days 7–19). (A) Vehicle-treated control mice showed no visible dermatitis-like lesions. (B) Disease-control mice exposed to DNCB and treated with vehicle showed persistent erythema, dryness, edema, and inflammatory skin changes. (C) Oral prednisolone and (D) topical prednisolone attenuated the visible severity of AD-like lesions compared with the disease-control group. (E) Oral I3C and (F) topical I3C were associated with improvement of DNCB-induced ear and dorsal skin lesions, with visibly reduced inflammatory changes by day 19. **Abbreviations**: AD, atopic dermatitis; DNCB, 1-chloro-2,4-dinitrobenzene; I3C, indole-3-carbinol.

### *In silico* molecular docking analysis

Molecular docking analysis was performed following previously reported methodologies [16, 17] to evaluate the binding affinity of I3C with target cytokines IL-13 and IL-4. The three-dimensional structures of IL-13 (PDB ID: 5L6Y) and IL-4 (PDB ID: 5FHX) were retrieved from the Protein Data Bank. The ligand structure of I3C was obtained from the PubChem database and converted into Protein Data Bank, Partial Charge and Atom Type format (PDBQT) format. Protein structures underwent processing to remove water molecules and add polar hydrogens and Kollman charges. Docking simulations were conducted using AutoDock Vina. The grid box was centered at predefined coordinates covering the active binding region of each protein, with dimensions of 40 × 40 × 40 Å. The exhaustiveness parameter was set to 8. Default parameters were used for all other settings. Binding affinity was evaluated based on the lowest binding energy (kcal/mol), and conformations were ranked accordingly. Root mean square deviation (RMSD) values were also considered to assess the consistency of binding poses. The docked complexes were visualized using PyMOL.

### Ethical statement

Ethical approval for this study was obtained from the Research Ethics Institutional Board of LCWU, Lahore (ORIC/LCWU/105).

### Statistical analysis

The sample size (*n* ═ 6 animals per group) was determined based on previously published studies employing similar DNCB-induced AD models and established practices in exploratory animal studies [13]. All statistical analyses were performed using GraphPad Prism (version 8.2). Data are expressed as mean ± standard deviation (SD). Longitudinal outcomes, including ear and dorsal skin AD scores and ear thickness, were assessed using a mixed-effects two-way repeated-measures analysis of variance (ANOVA) followed by Tukey’s post hoc test. Day 7 measurements were considered post-induction baseline assessments prior to treatment initiation, and treatment effects were evaluated using a mixed-effects two-way repeated-measures ANOVA incorporating both day 7 and day 19 observations. The statistical approach accounted for time-dependent repeated observations. Where applicable, effect estimates along with 95% confidence intervals were reported for oral-versus-topical treatment comparisons in addition to *P*-values. Although individual AD severity categories were ordinal, overall AD clinical scores were analyzed using two-way repeated-measures ANOVA, consistent with previous experimental AD studies employing similar scoring systems. TLC, DLC, and RT-qPCR data were analyzed using one-way ANOVA followed by Tukey’s post hoc test. A *P*-value ≤ 0.05 was considered statistically significant. *P* ≤ 0.05, * *P* ≤ 0.01, and *** *P* ≤ 0.001.

## Results

### I3C ameliorated visible symptoms of AD

The AD score on dorsal skin and ear was determined on day 7 and day 19. The results for both days are as follows:

### I3C reduced ear AD clinical scores

On day 7, inflammation and redness were observed on the ears of mice in all groups, except the control group, following successful sensitization on days 0 and 3. The mean AD score on the ear was elevated in all groups compared to the control group (0.00 ± 0.00), confirming AD induction: diseased (1.83 ± 0.40), oral prednisolone (1.67 ± 0.52), topical prednisolone (1.50 ± 0.54), oral I3C (1.33 ± 0.51), and topical I3C (1.33 ± 0.51).

By day 19, the mean AD score on the ear of the diseased group (2.50 ± 0.54) remained elevated compared to the control group (0.00 ± 0.00). Clinical scores were significantly reduced in all treatment groups after administration of the reference drug (oral and topical prednisolone; 1.33 ± 0.52, each) and the test drug (oral I3C; 1.33 ± 0.52, and topical I3C; 0.83 ± 0.40), relative to the diseased group (2.50 ± 0.54) (Figures 2 and 3A).

**Figure 3.**
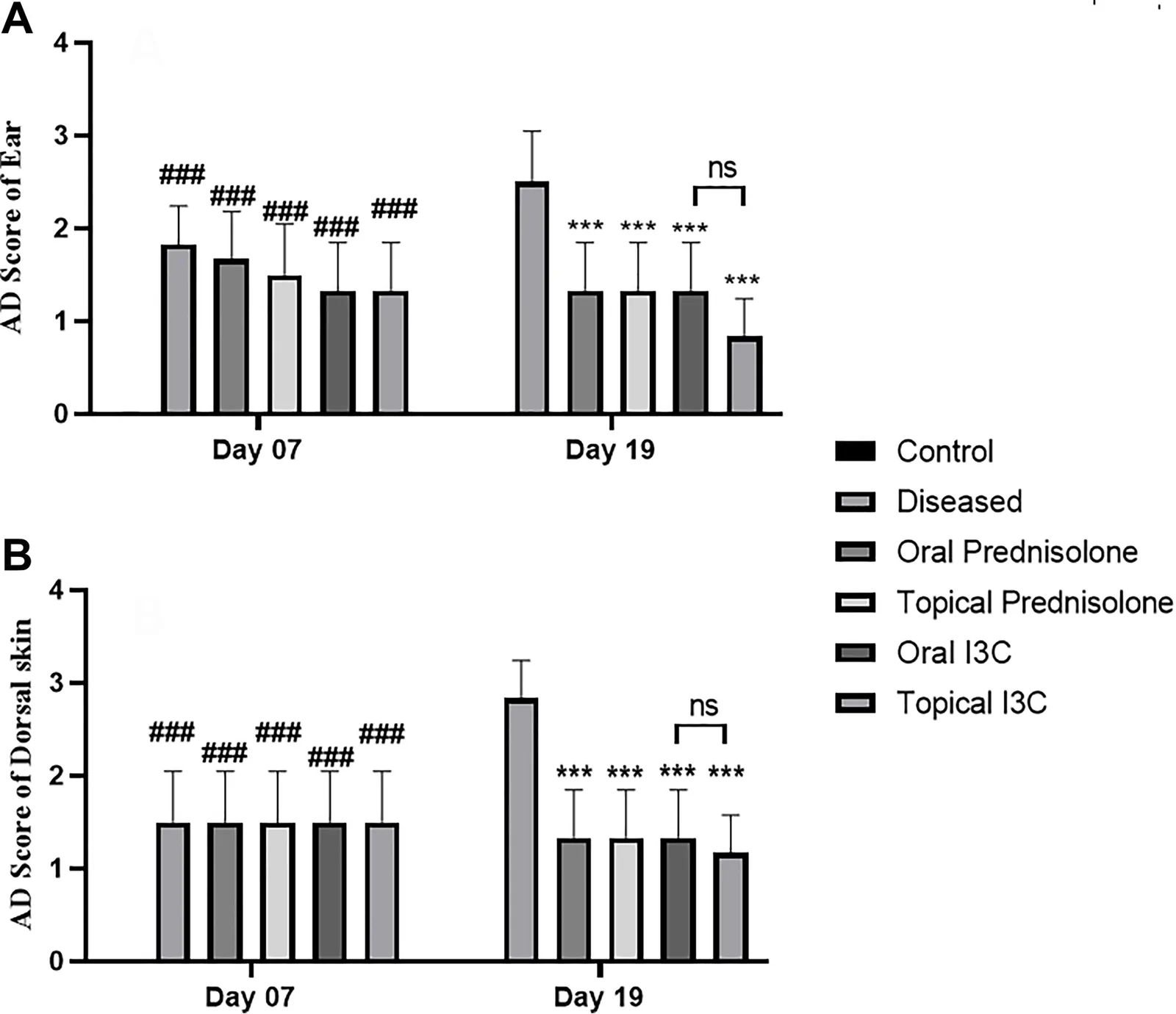
**Effect of I3C on clinical severity scores in DNCB-induced atopic dermatitis-like lesions.** AD clinical scores were assessed on day 7, after DNCB sensitization and before treatment initiation, and on day 19, after 13 consecutive days of treatment with oral or topical prednisolone or I3C. (A) Ear AD clinical score. (B) Dorsal skin AD clinical score. Control animals had a score of 0.00 ± 0.00; therefore, no visible bar is shown for the control group. Data are presented as mean ± SD. ###*P <* 0.001 versus the control group; ***P < 0.001 versus the diseased group. The bracket indicates the comparison between oral and topical I3C treatment; ns indicates a non-significant difference. **Abbreviations**: AD, atopic dermatitis; DNCB, 1-chloro-2,4-dinitrobenzene; I3C, indole-3-carbinol; ns, non-significant; SD, standard deviation.

For ear AD clinical score, a two-way repeated-measures ANOVA revealed a significant effect of treatment group (*P* < 0.0001). A significant interaction between treatment and time was also observed (*P* ═ 0.0181), indicating that disease progression over time varied among untreated and treatment-receiving groups. In contrast, time alone did not produce a significant overall effect (*P* ═ 0.5474; 95% CI: −0.1309 to 0.2420). These findings suggest that prednisolone- and I3C-treated groups exhibited lower AD severity scores over time compared with untreated diseased mice; however, these findings should be interpreted as exploratory.

### I3C reduced dorsal skin AD clinical scores

Following two sensitizations on days 0 and 3, inflammation and erythema were observed on the dorsal skin of mice in all experimental groups, excluding the control group, by day 7. The mean AD score on the dorsal skin of these groups (1.50 ± 0.54 each) was significantly elevated compared to the control group (0.00 ± 0.00), confirming successful AD induction.

By day 19, the mean AD score in the diseased group (2.83 ± 0.41) remained elevated relative to the control group (0.00 ± 0.00). Treatment with reference drugs (oral and topical prednisolone; 1.33 ± 0.52 each) and test drugs (oral I3C; 1.33 ± 0.52, and topical I3C; 1.17 ± 0.41) significantly reduced the clinical score (*P* < 0.001) compared to the diseased group (2.83 ± 0.41) (Figures 2 and 3B).

For dorsal skin AD clinical scores, a two-way repeated-measures ANOVA revealed a significant effect of treatment group (*P* < 0.0001) and a significant interaction between treatment and time (*P* ═ 0.0001). This interaction suggests that the trajectory of AD severity over time differed significantly between untreated and treated groups. At day 19, oral and topical prednisolone demonstrated comparable effects on dorsal skin AD scores (mean difference = 0.000; 95% CI: −0.7962 to 0.7962; adjusted *P* > 0.9999). Similarly, no significant difference was observed between oral and topical I3C (mean difference = 0.1667; 95% CI: −0.6295 to 0.9628; adjusted *P* ═ 0.9894). However, the main effect of time alone was not statistically significant (*P* ═ 0.3729; 95% CI: −0.2715 to 0.1048). These preliminary findings suggest that prednisolone- and I3C-treated groups exhibited lower dorsal skin AD severity scores compared to untreated diseased mice, aligning with observations from ear AD clinical scores.

### I3C reduced ear thickness

At day 7, following DNCB sensitization on days 0 and 3, ear thickness was significantly increased in all experimental groups—diseased (2.51 ± 0.16), oral prednisolone (2.10 ± 0.14), topical prednisolone (2.08 ± 0.18), oral I3C (2.11 ± 0.16), and topical I3C (1.82 ± 0.15)—when compared to the control group (1.00 ± 0.20).

By day 19, ear thickness remained elevated in the diseased group (2.39 ± 0.17) relative to the control group (1.10 ± 0.13). Conversely, all treatment groups exhibited significantly reduced ear thickness (*P* < 0.001) following administration of I3C (oral I3C; 1.73 ± 0.23, topical I3C; 1.61 ± 0.24) and prednisolone (oral prednisolone; 1.93 ± 0.15, topical prednisolone; 1.46 ± 0.13). A significant difference was observed between oral and topical prednisolone administration (*P* < 0.001), while oral and topical I3C treatments yielded comparable results.

A two-way repeated-measures ANOVA for ear thickness demonstrated significant effects of treatment group (*P* < 0.0001), time (*P* < 0.0001), and a treatment × time interaction (*P* ═ 0.0001). The mean difference in ear thickness between day 7 and day 19 was 0.2319 (95% CI: 0.1567–0.3072). These findings suggest distinct ear-thickness trajectories between untreated and treatment-receiving groups over the experimental period. However, given baseline differences observed at post-induction assessment, these findings should be considered exploratory.

Overall, the ear thickness results were consistent with the AD clinical scoring, both indicating progression of inflammation in untreated diseased mice and attenuation of disease severity in treatment-receiving groups (Figure 4).

**Figure 4.**
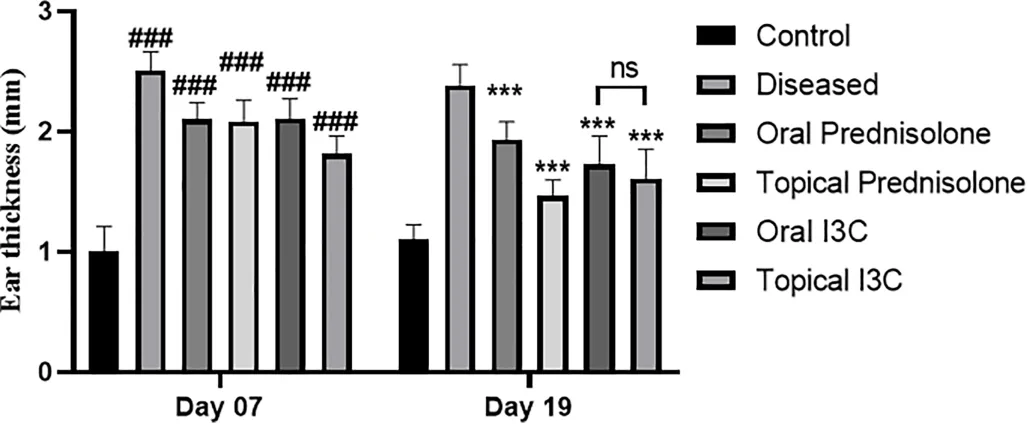
**Effect of I3C on ear thickness in DNCB-induced atopic dermatitis-like lesions.** Ear thickness was measured on day 7, after DNCB sensitization and before treatment initiation, and on day 19, after 13 consecutive days of oral or topical treatment with prednisolone or I3C. DNCB exposure increased ear thickness on day 7 compared with the control group, confirming successful induction of AD-like inflammation. On day 19, treatment with prednisolone or I3C reduced ear thickness compared with the diseased group. Oral and topical I3C produced comparable effects, as indicated by the non-significant difference between these groups. Data are presented as mean ± SD. ###*P <* 0.001 versus the control group; ***P < 0.001 versus the diseased group. The bracket indicates the comparison between oral and topical I3C treatment; ns indicates a non-significant difference. **Abbreviations**: AD, atopic dermatitis; DNCB, 1-chloro-2,4-dinitrobenzene; I3C, indole-3-carbinol; ns, non-significant; SD, standard deviation.

**Figure 5.**
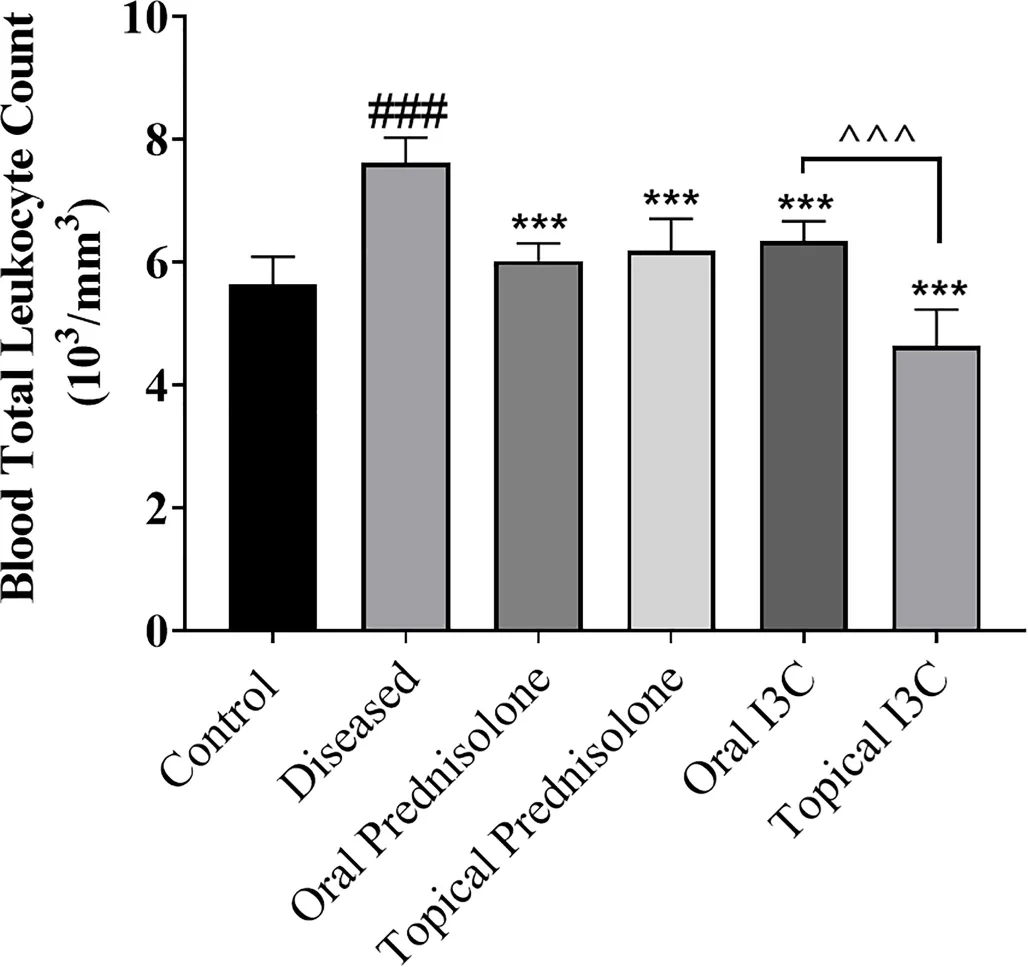
**Effect of I3C on total leukocyte count in DNCB-induced atopic dermatitis.** Blood TLC was evaluated after treatment to assess systemic inflammatory changes in control, diseased, prednisolone-treated, and I3C-treated mice. The diseased group showed a significant increase in TLC compared with the control group, whereas oral and topical prednisolone and I3C treatments significantly reduced TLC compared with the diseased group. Topical I3C produced a greater reduction in TLC than oral I3C. Data are presented as mean ± SD. ###*P <* 0.001 versus the control group; ***P < 0.001 versus the diseased group; ˆˆˆ*P <* 0.001 for comparison between oral and topical I3C treatment. **Abbreviations**: DNCB, 1-chloro-2,4-dinitrobenzene; I3C, indole-3-carbinol; SD, standard deviation; TLC, total leukocyte count.

### Effect of I3C on hematological parameters

#### I3C treatment reduced total leukocyte counts

Values in the diseased group (7.617 ± 0.41) were elevated compared to the control group (5.633 ± 0.45). These counts were significantly reduced (*P* < 0.001) in treatment groups following administration of I3C (oral I3C; 6.333 ± 0.33, topical I3C; 4.633 ± 0.59) and prednisolone (oral prednisolone; 6.017 ± 0.28, topical prednisolone; 6.183 ± 0.52). Notably, topical I3C resulted in a significantly greater reduction in total leukocyte counts compared to oral I3C (Figure 5).

#### Effects of I3C treatment on differential leukocyte counts

Lymphocyte levels in the diseased group (84.17 ± 2.04%) were significantly increased compared to the control group (79.50 ± 2.43%). Following treatment, lymphocyte levels were significantly reduced in the oral prednisolone (71.17 ± 1.47%), topical prednisolone (77.33 ± 1.63%), and topical I3C (75.67 ± 4.27%) groups compared to the diseased group. While the oral I3C group (81.00 ± 1.78%) showed a numerically lower mean lymphocyte level than the diseased group, this difference was not statistically significant. Topical I3C produced a significantly greater (*P* < 0.01) reduction in lymphocyte levels than oral I3C (Figure 6A).

**Figure 6.**
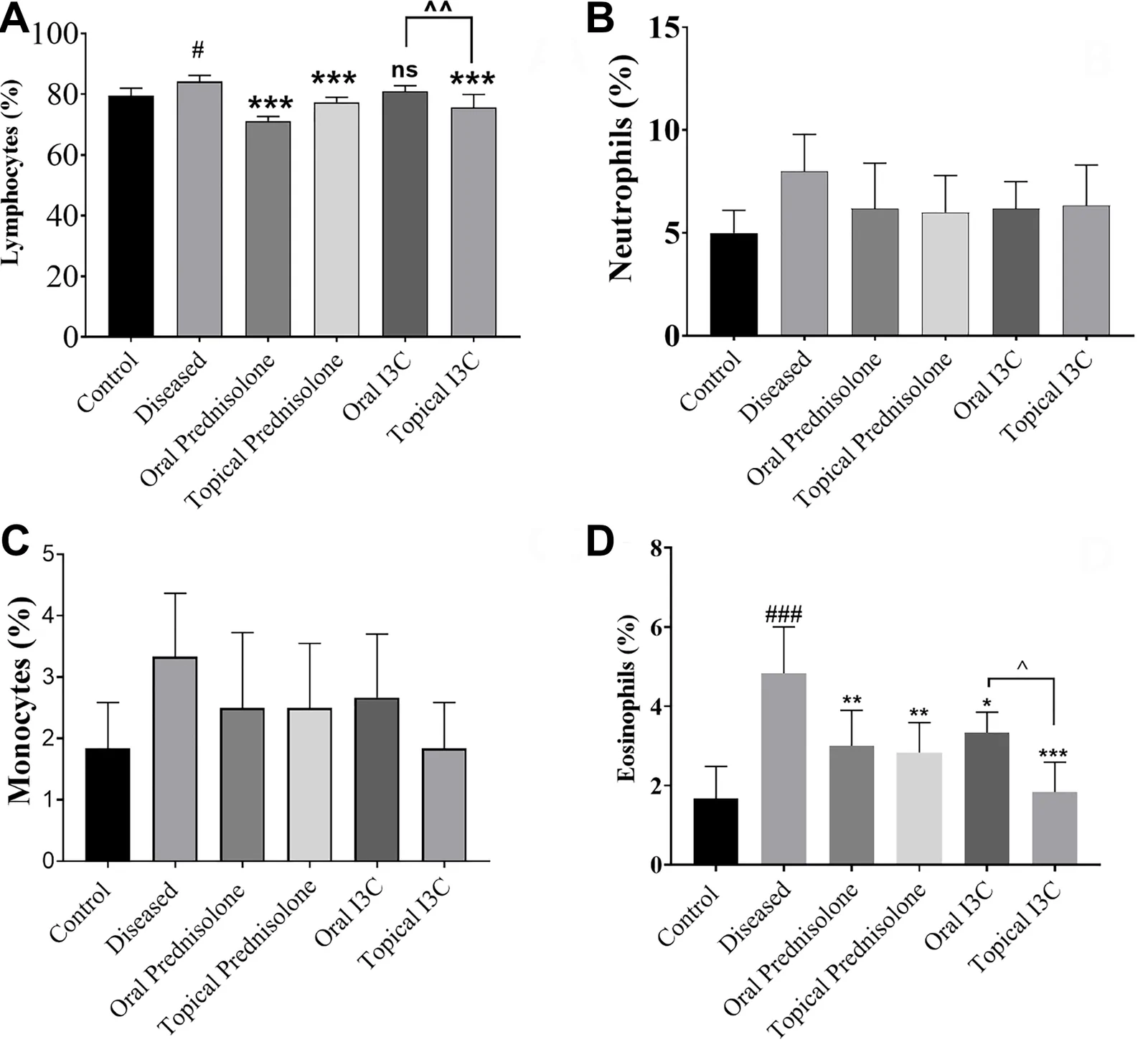
**Effect of I3C on differential leukocyte counts in DNCB-induced atopic dermatitis.** Blood differential leukocyte counts were analyzed after treatment in control, diseased, prednisolone-treated, and I3C-treated mice. (A) Lymphocyte percentage was increased in the diseased group compared with the control group and was reduced after oral prednisolone, topical prednisolone, and topical I3C treatment; oral I3C showed a non-significant reduction compared with the diseased group. (B) Neutrophil percentage showed a numerical increase in the diseased group and a downward trend in treatment groups. (C) Monocyte percentage was numerically increased in the diseased group and reduced mainly after topical I3C treatment. (D) Eosinophil percentage was increased in the diseased group and significantly reduced after prednisolone and I3C treatment, with topical I3C producing a greater reduction than oral I3C. Data are presented as mean ± SD. #*P <* 0.05 and ###*P <* 0.001 versus the control group; *P < 0.05, **P < 0.01, and ***P < 0.001 versus the diseased group; ˆ*P <* 0.05 and ˆˆ*P <* 0.01 for comparison between oral and topical I3C treatment. ns indicates a non-significant difference. **Abbreviations**: DNCB, 1-chloro-2,4-dinitrobenzene; I3C, indole-3-carbinol; ns, non-significant; SD, standard deviation.

Neutrophil levels in the diseased group (8.00 ± 1.79%) were also elevated compared to the control group (5.00 ± 1.09%). These values were reduced in treatment groups following I3C administration (oral I3C; 6.17 ± 1.33%, topical I3C; 6.33 ± 1.97%) and prednisolone administration (oral prednisolone; 6.17 ± 2.23%, topical prednisolone; 6.00 ± 1.79%). Topical and oral I3C groups exhibited comparable results (Figure 6B).

Monocyte values in the diseased group (3.33 ± 1.03%) were increased compared to the control group (1.83 ± 0.75%). These values were reduced in treatment groups following I3C administration (oral I3C; 2.67 ± 1.03%, topical I3C; 1.83 ± 0.75%) and prednisolone administration (oral prednisolone; 2.50 ± 1.23%, topical prednisolone; 2.50 ± 1.05%). Although the mean monocyte values in the topical I3C group were generally lower than in the oral I3C group, the differences were not statistically significant (Figure 6C).

Eosinophil values in the diseased group (4.83 ± 1.16%) were increased compared to the control group (1.67 ± 0.82%). These values were significantly reduced in treatment groups following I3C administration (oral I3C; 3.33 ± 0.52%, topical I3C; 1.83 ± 0.75%) and prednisolone administration (oral prednisolone; 3.00 ± 0.89%, topical prednisolone; 2.83 ± 0.75%). The topical I3C group demonstrated a significantly greater reduction (*P* < 0.05) compared to the oral I3C group (Figure 6D).

### I3C ameliorates IL-4 and IL-13 expression levels

The expression levels of IL-4 and IL-13 were quantified using RT-qPCR.

### I3C reduces IL-4 expression levels

IL-4 levels were upregulated in the diseased group (3.94 ± 0.55) compared to the control group (1.02 ± 0.26). These levels were significantly reduced in all treatment groups (all *P* < 0.001) following administration of I3C (oral I3C; 0.99 ± 0.29, topical I3C; 0.48 ± 0.28) and prednisolone (oral; 1.493 ± 0.82, topical; 1.24 ± 0.34). Although the mean IL-4 expression was numerically lower in the topical I3C group, the difference between oral and topical administration was not statistically significant (Figure 7A).

**Figure 7.**
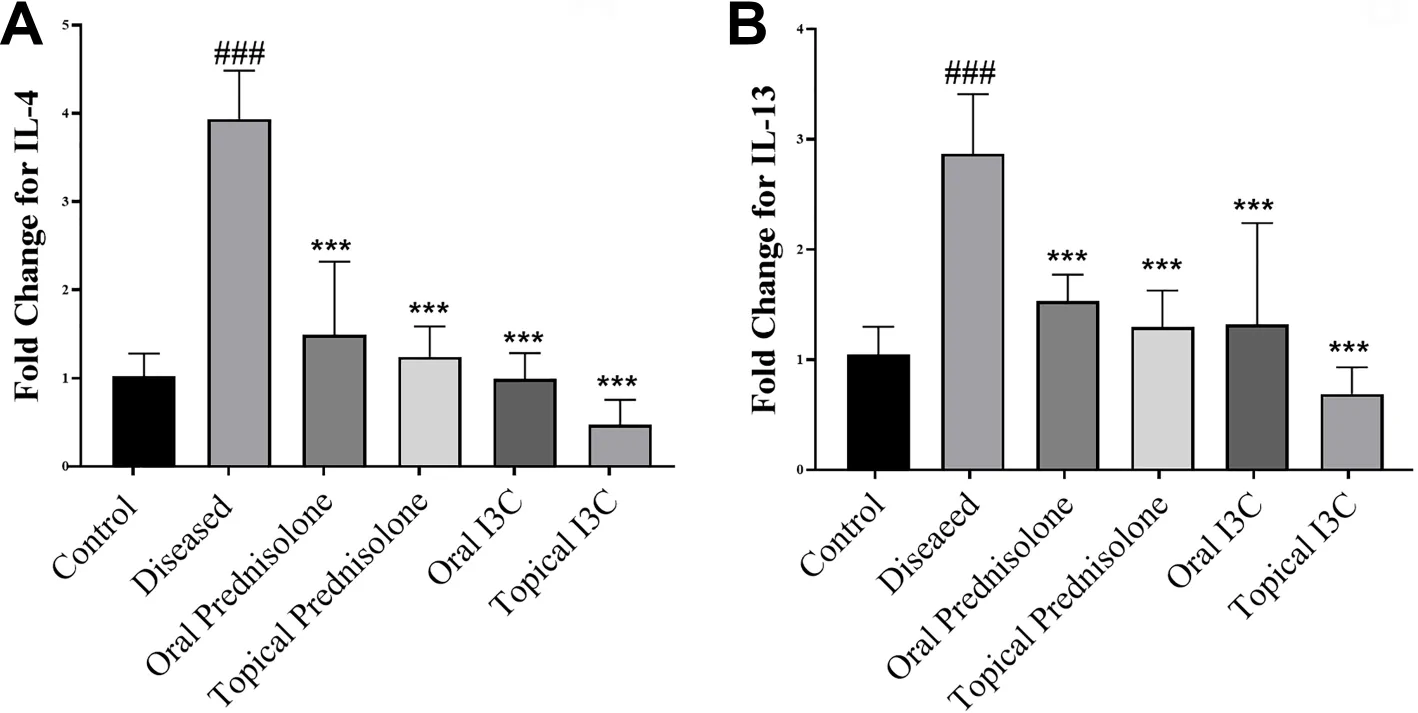
**Effect of I3C on IL-4 and IL-13 mRNA expression in DNCB-induced atopic dermatitis.** Cytokine mRNA expression was quantified in ear tissue by RT-qPCR after treatment with oral or topical prednisolone or I3C. (A) IL-4 expression was markedly increased in the diseased group compared with the control group and was significantly downregulated after prednisolone and I3C treatment. (B) IL-13 expression was similarly increased in the diseased group and significantly reduced after oral and topical treatment, with topical I3C showing the lowest mean expression level. Data are presented as mean ± SD. ###*P <* 0.001 versus the control group; ***P < 0.001 versus the diseased group. **Abbreviations**: DNCB, 1-chloro-2,4-dinitrobenzene; I3C, indole-3-carbinol; IL-4, interleukin-4; IL-13, interleukin-13; mRNA, messenger RNA; RT-qPCR, quantitative real-time polymerase chain reaction; SD, standard deviation.

### I3C downregulated IL-13 mRNA expression

IL-13 levels were also significantly elevated in the diseased group (2.87 ± 0.54) compared to the control group (1.04 ± 0.25). These levels were significantly reduced in all treatment groups (all *P* < 0.001) following administration of I3C (oral I3C; 1.32 ± 0.92, topical I3C; 0.69 ± 0.24) and prednisolone (oral; 1.53 ± 0.24, topical; 1.29 ± 0.33). While the mean IL-13 expression was numerically lower in the topical I3C group, the difference between oral and topical administration was not statistically significant (Figure 7B).

### Effect of I3C on histopathological parameters of dorsal skin tissue

Histopathological evaluation of dorsal skin sections, performed by an independent histopathologist using an Olympus CX31 microscope, revealed that the diseased group exhibited epidermal hyperplasia with irregular epidermal thickening and areas of surface crust formation compared to the control group. Mild inflammatory cell infiltration was also observed within the dermis (Figure 8B).

**Figure 8.**
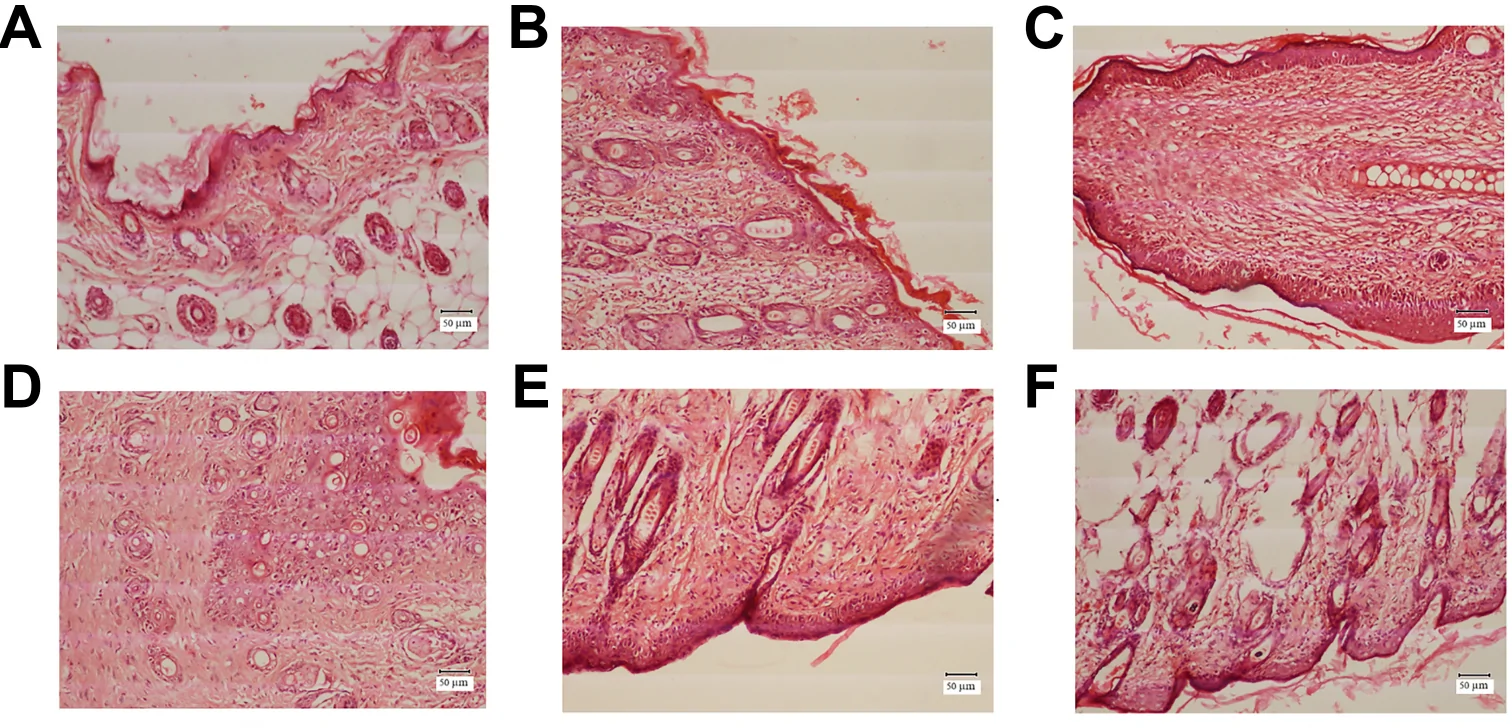
**Histopathological evaluation of dorsal skin in DNCB-induced atopic dermatitis after I3C treatment.** Representative photomicrographs of H&E-stained dorsal skin sections showing epidermal and dermal changes across experimental groups. (A) Control group showing preserved skin architecture with normal epidermal morphology and no apparent inflammatory cell infiltration. (B) Diseased group showing AD-like histopathological alterations, including epidermal hyperplasia, irregular epidermal thickening, surface crusting, and dermal inflammatory cell infiltration. (C) Oral prednisolone and (D) topical prednisolone groups showing reduced epidermal alterations and inflammatory cell infiltration compared with the diseased group. (E) Oral I3C and (F) topical I3C groups showing improvement in DNCB-induced histopathological changes, with less apparent epidermal thickening and dermal inflammatory cell infiltration; the topical I3C group showed skin morphology qualitatively closer to the control group. Images were captured at 10 × 10 magnification. Scale bars = 50 µm. **Abbreviations**: AD, atopic dermatitis; DNCB, 1-chloro-2,4-dinitrobenzene; H&E, hematoxylin and eosin; I3C, indole-3-carbinol.

Histopathological examination of dorsal skin sections from the prednisolone- and I3C-treated groups showed qualitatively less apparent epidermal alterations and inflammatory cell infiltration compared to the diseased group. The topical I3C group displayed skin morphology that qualitatively appeared closer to the control group (Figure 8C–F).

### Molecular docking

Molecular docking simulations demonstrated that I3C exhibited binding affinity toward both IL-4 and IL-13. For Interleukin-4, the optimal binding pose showed an affinity of −5.1 kcal/mol, with multiple conformations exhibiting similar binding energies (−5.1 to −4.6 kcal/mol). For Interleukin-13, the lowest binding energy observed was -5.3 kcal/mol, indicating slightly stronger binding compared to IL-4. Other conformations ranged from −4.9 to −4.3 kcal/mol, suggesting stable ligand–protein interactions. The docked conformations exhibited low RMSD values (< 2–3 Å) for several poses, indicating consistent binding orientations of I3C within the target sites. Overall, I3C demonstrated moderate binding affinity toward both cytokines, with relatively consistent docking scores across multiple poses (Figure 9).

**Figure 9.**
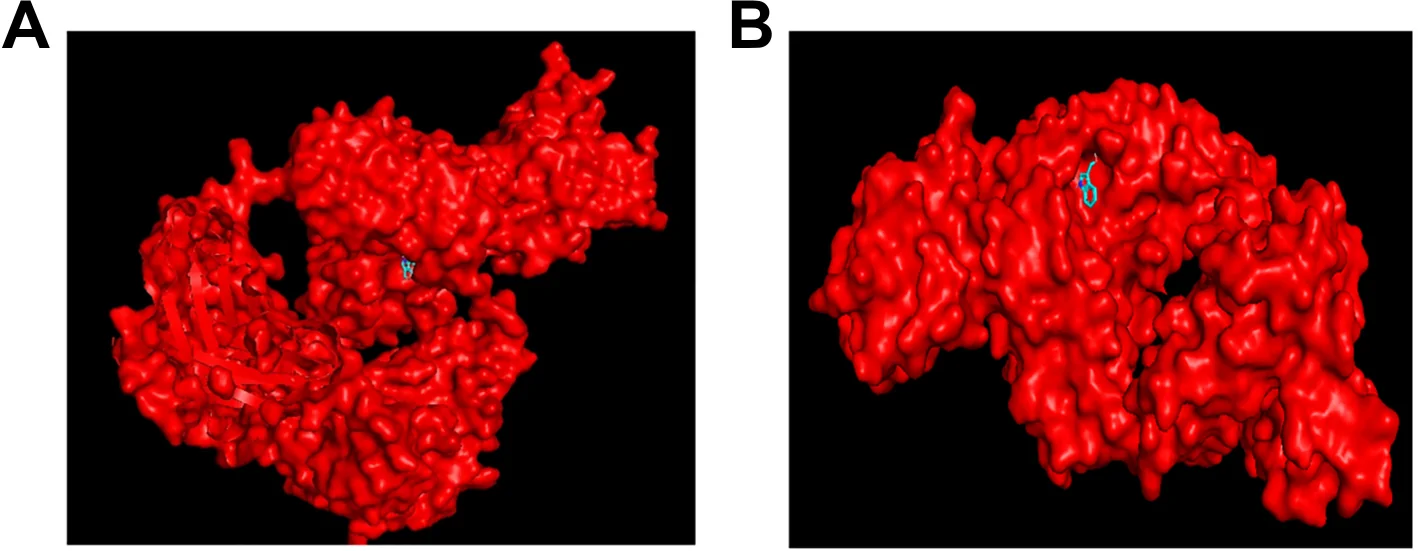
**Molecular docking of I3C with IL-4 and IL-13.** Surface representation of the predicted binding poses of I3C with target cytokines involved in atopic dermatitis-associated inflammatory responses. (A) Docked complex of I3C with IL-4 showing a predicted binding energy of -5.1 kcal/mol. (B) Docked complex of I3C with IL-13 showing a predicted binding energy of −5.3 kcal/mol. These findings indicate moderate predicted ligand–protein interactions, with slightly stronger binding affinity of I3C toward IL-13 than IL-4. **Abbreviations**: I3C, indole-3-carbinol; IL-4, interleukin-4; IL-13, interleukin-13.

## Discussion

This study investigated the therapeutic potential of I3C in AD by evaluating its effects on clinical scores, ear and dorsal skin thickness, and hematological parameters such as TLC and DLC. We also examined I3C’s modulation of regional mRNA expression of IL-4 and IL-13 through qPCR and in silico ligand-protein interaction studies. Histopathological analysis explored changes in skin architecture induced by AD and subsequent modulation by I3C.

Our preliminary findings suggest that I3C holds promise for experimental AD. Treatment with I3C in the disease group attenuated AD clinical scores, ear and dorsal skin thickness, and total leukocyte count, with variable changes in DLC parameters. Furthermore, regional molecular expression data revealed that I3C administration downregulated IL-4 and IL-13 mRNA expression. Molecular docking analysis provided computational evidence of potential interactions between I3C and these cytokines, demonstrating moderate and consistent binding affinities. Histopathological findings qualitatively supported these observations, suggesting reduced inflammatory cell infiltration and epidermal alterations after I3C treatment.

These findings align with a recent study demonstrating I3C’s protective effects in experimental AD [12]. While that study highlighted I3C’s anti-inflammatory potential in skin inflammation, our work extends these observations by comparing oral and topical administration routes and specifically evaluating IL-4 and IL-13 mRNA expression, supported by molecular docking analysis. Although both oral and topical I3C yielded broadly comparable outcomes, topical administration showed numerically greater improvement in several parameters; however, most oral-versus-topical comparisons lacked statistical significance. Due to the absence of topical vehicle in oral-treatment groups and oral vehicle in topical-treatment groups, comparisons between administration routes should be interpreted cautiously. Further research is necessary to determine if the route of administration influences therapeutic efficacy.

AD is a chronic, pruritic, inflammatory skin condition prone to relapse at any age [17]. Its incidence is rapidly increasing in developing nations [18]. Research indicates that AD is a multifaceted disease with distinct subtypes, similar to other allergic conditions like asthma [19]. It is characterized by sensitive, dry skin, isolated eczematous lesions, and intense itching. A hallmark of atopic diseases is IgE-mediated allergic reactivity to environmental allergens [20].

AD is a complex disease with multiple etiologies, significantly impacting patients’ and their families’ quality of life, and posing substantial social and economic burdens [21]. AD disrupts sleep cycles, hindering daily functioning [18]. It is part of the atopic disorder hierarchy, encompassing allergic rhinitis, asthma, and food intolerance, all significant comorbidities. Approximately 80% of AD cases manifest in infancy or childhood, with the remainder emerging in adulthood [20].

Current AD treatments primarily include topical and oral corticosteroids, calcineurin inhibitors, and recently approved phosphodiesterase 4 (PDE4) inhibitors. UV light therapy is also recommended for severe AD [20]. Newer medications specifically target cytokines, such as dupilumab, which targets the interleukin pathway [19, 21]. However, these therapies are often associated with adverse effects. Consequently, natural substances are gaining interest among researchers due to their lower toxicity and comparable therapeutic activities [4].

The documented health benefits of cruciferous vegetable consumption are often attributed to the antioxidant and anti-inflammatory activities of glucosinolates and their metabolites, such as I3C and sulforaphane. These metabolites have been reported to offer therapeutic benefits in neurological diseases like depression, autism, and schizophrenia, and aid in treating musculoskeletal disorders. Improvements in blood sugar levels, lipid profiles, and blood pressure have also been associated with glucosinolate metabolites [22]. Clinical trials suggest a strong correlation between cruciferous vegetable intake and a reduced risk of breast cancer [23].

I3C exhibits low toxicity and participates in various cellular and molecular activities, particularly carcinogen metabolism. This compound is a prospective clinical drug capable of inducing cell cycle arrest or apoptosis and enhancing the synthesis of DNA repair proteins [24]. Due to its antioxidant, anti-inflammatory, and anti-apoptotic properties, and its potential to restore normal endocrine system activities, I3C may be a viable therapeutic agent for conditions such as diabetes, cancer, cardiotoxicity, and endocrine problems [23]. I3C is known to reduce prostate, colon, breast, and liver cancers [22]. Preliminary clinical investigations indicate I3C possesses anticancer, antibacterial, antiviral, and anti-inflammatory properties [24].

In this study, we utilized an AD mouse model induced by DNCB acetone solution to evaluate I3C’s anti-inflammatory activity. DNCB-induced AD-like lesions in mice are well-established to resemble human AD characteristics [25].

Our findings indicate that I3C reduced AD symptoms, including dry and scraping skin, redness, and edema. On the final treatment day, I3C-treated groups exhibited milder symptoms compared to the diseased group, consistent with previous studies reporting attenuation of AD-like symptoms in experimental models [2, 5]. The diseased group showed increased ear and epidermal thickness and inflammatory cell infiltration. In contrast, qualitative histopathological examination of I3C-treated groups suggested less apparent epidermal alterations and inflammatory cell infiltration. These qualitative histopathological changes align with a potential ameliorative effect of I3C in AD and are consistent with previous studies demonstrating I3C’s anti-inflammatory effects in various inflammatory models [10, 11]. Future studies could incorporate quantitative histopathological scoring of epidermal thickness and inflammatory cell infiltration to strengthen histological evaluation.

Neutrophils, as the initial leukocytes to egress from circulation to sites of inflammation or infection, signify innate immune activation against invading pathogens. Monocytes and macrophages, crucial components of the innate immune system, are implicated in the pathogenesis of numerous immune-mediated diseases. Their infiltration into damaged tissues characterizes several autoimmune disorders [26]. The TLC and DLC levels were observed in the diseased group [27]. Treatment with I3C and prednisolone significantly reduced TLC. While differential leukocyte parameters exhibited varied responses, with some showing significant reductions and others demonstrating numerical downward trends, these findings align with prior research indicating I3C’s association with improved hematological and inflammatory parameters in experimental models [11].

AD is recognized as an inflammatory condition characterized by a Th2 cell-dominant immune response that progresses to a chronic phase. Th2-derived cytokines and inflammatory mediators, such as those secreted by mast cells and other innate immune cells, contribute to the pathogenesis of cutaneous inflammation in AD lesions [21]. Th2 cells induce significant inflammatory responses by secreting cytokines like IL-4 and IL-13. Elevated levels of these cytokines decrease the expression of proteins such as filaggrin and loricrin in keratinocytes, exacerbating epidermal barrier dysfunction [28]. Keratinocytes upregulate IL-4 and IL-13 receptors and release eosinophils in response to these cytokines. IL-4 and IL-13 have been shown to directly activate itch-sensory neurons, inducing pruritus, in addition to regulating IgE antibody production in B cells. Th2 cells also release IL-31, another pruritus-inducing cytokine [28]. In the present study, IL-4 and IL-13 expression levels were evaluated in a DNCB-induced AD mouse model. Results indicated elevated levels in the diseased group, which I3C treatment attenuated. The amelioration of cytokine levels may be attributed to I3C’s immunomodulatory and anti-inflammatory properties.

Molecular docking analysis revealed that I3C exhibits binding affinity towards IL-4 and IL-13, with binding energies of -5.1 kcal/mol and -5.3 kcal/mol, respectively, indicative of moderate interactions with both target cytokines. The slightly higher affinity for IL-13 suggests a comparatively stronger interaction, though the difference remains minimal. Multiple docking conformations with comparable binding energies were observed, indicating that I3C can adopt stable orientations within the binding regions of both IL-4 and IL-13. Furthermore, several poses exhibited low RMSD values (< 2–3 Å), reflecting consistency in binding orientation across predicted conformations.

These docking findings are consistent with the RT-qPCR results from the current study, where I3C treatment downregulated IL-4 and IL-13 expression levels. While molecular docking does not establish direct functional inhibition, the observed binding interactions support the possibility of I3C associating with these cytokines. Overall, the docking analysis provides supportive computational evidence aligning with the experimentally observed attenuation of IL-4 and IL-13 expression, without implying direct mechanistic inhibition.

Although topical I3C numerically enhanced some parameters more effectively, most differences between oral and topical administration were not statistically significant. The primary advantage of topical delivery is the avoidance of first-pass metabolism. Additionally, topical drug delivery bypasses the risks and inconveniences of oral and intravenous therapies, as well as variations in absorption due to factors like pH, enzymatic presence, and gastric emptying time [29].

The current study has several limitations. It did not explore a range of I3C doses, and only two inflammatory cytokines, IL-4 and IL-13, were evaluated, despite the involvement of numerous other inflammatory cytokines in AD pathogenesis. Therefore, future research should investigate additional inflammatory markers to further elucidate I3C’s anti-inflammatory activity. Moreover, this was not a mechanistic study; thus, the precise molecular pathways underlying I3C’s therapeutic effects were not investigated. The relatively small sample size (*n* ═ 6 animals per group) may also limit statistical power for certain hematological, molecular, and histopathological outcomes. Consequently, findings should be interpreted cautiously and warrant confirmation in future studies with larger sample sizes. Furthermore, AD clinical scores were based on an ordinal scoring system. While repeated-measures ANOVA was employed to assess treatment- and time-related effects, findings derived from these scores should be considered exploratory and interpreted with appropriate caution. Although animals were randomly allocated to treatment groups, some differences in disease severity were present at the Day 7 post-induction assessment. However, treatment-related conclusions were based on longitudinal changes over time rather than Day 7 values alone. Nevertheless, these baseline differences may influence the interpretation of treatment effects and should be considered. While qualitative histopathological assessment and clinical scoring were performed in a blinded manner, formal blinding was not implemented for all outcome measures, which should be considered when interpreting the findings. Future studies incorporating quantitative histological scoring could further strengthen histopathological evaluation. Additionally, RT-qPCR normalization was performed using a single reference gene (GAPDH). Future studies incorporating multiple validated reference genes and evaluating reference-gene stability may enhance the robustness of gene expression analyses.

## Conclusion

In conclusion, this exploratory study suggests that Indole-3-carbinol treatment attenuated AD clinical scores of the ears and dorsal skin, reduced ear thickness, and induced accompanying changes in hematological parameters in a DNCB-induced AD model. Furthermore, expression analysis revealed that Indole-3-carbinol administration downregulated IL-4 and IL-13 mRNA expression, complemented by molecular docking studies demonstrating moderate binding affinities and potential interactions between I3C and IL-4/IL-13 proteins. Qualitative histopathological findings were consistent with these observations, suggesting less apparent inflammatory cell infiltration and epidermal alterations. However, further studies evaluating dose-response relationships, protein-level cytokine expression, serum IgE, pruritus-related outcomes, and skin-barrier function are warranted to further characterize the therapeutic potential of I3C in AD.

## Data Availability

Data are available from the corresponding authors upon reasonable request
